# Mesalazine-induced myocarditis: a case report

**DOI:** 10.1186/s13256-017-1557-z

**Published:** 2018-02-22

**Authors:** Shiva T. Radhakrishnan, Aruchuna Mohanaruban, Sami Hoque

**Affiliations:** 1 Imperial College Healthcare, St. Mary’s Hospital, London, UK; 2 Barts Health NHS Trust, Whipps Cross University Hospital, London, UK

**Keywords:** Myocarditis, Chest pain, Mesalazine, Ulcerative colitis, CMR

## Abstract

**Background:**

Myocarditis is a rare complication of therapy with mesalazine, a drug widely prescribed in the treatment of inflammatory bowel disease.

**Case presentation:**

We report a case of myocarditis occurring in a 49-year-old British man 10 days following initiation of mesalazine therapy for treatment of ulcerative colitis. He presented with troponin-positive chest pain, and the diagnosis of myocarditis was confirmed on the basis of cardiac magnetic resonance imaging, which showed subepicardial delayed gadolinium enhancement in the basal to middle inferior and inferolateral segments of the heart. The patient’s symptoms and condition improved upon stopping mesalazine, and he made a full recovery.

**Conclusions:**

Mesalazine-induced myocarditis may be more common than first appreciated and is potentially fatal. Therefore, it is imperative that clinicians be aware of this potentially life-threatening adverse effect of mesalazine therapy and warn patients to seek urgent medical attention if cardiac symptoms arise.

## Background

Mesalazine (5-ASA) is commonly prescribed as first-line medical therapy in the treatment of ulcerative colitis (UC) [1]. It is often well tolerated by patients, with the most frequently reported side effects being nausea, vomiting, and abdominal pain [2]. Rarer side effects include pancreatitis, blood dyscrasias, and cardiovascular problems [3]. The exact mechanism of its action is unknown, but it is thought to interact with the damaged epithelium seen in inflammatory bowel disease (IBD) [4]. Various theories exist regarding the mechanism of action of 5-ASA, including inhibition of interleukin-2 production, thereby decreasing T-cell proliferation [5].

Myocarditis is a rare but recognized complication of 5-ASA therapy and can potentially evolve to cause cardiogenic shock and death [6]. The etiology by which 5-ASAs are said to cause myocarditis is unclear, but it is postulated to be cell-mediated through a hypersensitivity reaction. We describe the case of a 49-year-old man presenting with probable 5-ASA-induced myocarditis.

## Case presentation

A 49-year-old British man presented to our hospital with an acute flare of UC, describing a 2-week history of bloody diarrhea. He was initially prescribed both oral and topical 5-ASA therapy and responded well to therapy, reporting an improvement in his abdominal symptoms. However, 10 days after commencing 5-ASA therapy, he was readmitted with central chest pain, pleuritic in nature and associated with severe dyspnea. The patient’s cardiovascular and respiratory examinations were unremarkable, and he was normotensive and apyrexial. His electrocardiogram demonstrated sinus tachycardia, and his cardiac enzymes were elevated, with a troponin T level of 146 ng/ML. He was treated for an acute coronary syndrome with dual-antiplatelet therapy, and an urgent inpatient angiogram was scheduled.

**Fig. 1:**
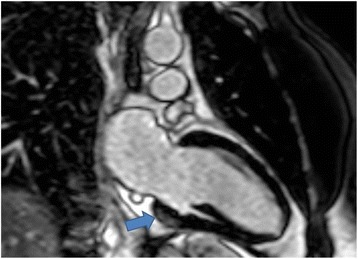
Cardiac magnetic resonance imaging of patient showing myocarditis. The arrow shows sub epicardial delayed gadolinium enhancement in the basal to inferolateral segment of the heart

The coronary angiogram revealed smooth, unobstructed coronary arteries, and no cause was identified to account for the patient’s chest pain and troponin rise. Subsequently, cardiac magnetic resonance imaging (CMR) was performed, which showed subepicardial delayed gadolinium enhancement in the basal to middle inferior and inferolateral segments of the heart (Fig. 1), with matching high signal intensity seen on T2-weighted images of the same area. The left ventricle was nondilated with preserved systolic function. On the basis of these CMR findings, the patient was diagnosed with acute myocarditis, and the etiology for this was considered, including viral myocarditis, extraintestinal manifestation of IBD, a vasculitic or autoimmune process, or a drug-induced myocarditis. The patient had no history of a prodromal illness to suggest an underlying infection, and he was systemically well, making a vasculitic or autoimmune process less likely. Extraintestinal symptoms of IBD usually occur years following diagnosis, whereas our patient’s symptoms developed soon after starting a new drug therapy, which suggested that 5-ASA was the culprit.

The patient was treated conservatively with analgesia, and 5-ASA was promptly stopped. A working diagnosis of 5-ASA-induced myocarditis was supported by the gradual improvement in the patient’s condition following cessation of the drug, and he was discharged to home 3 days later. Follow-up CMR was performed after 2 months, and this showed no myocardial edema as well as reduced gadolinium enhancement of the epicardium and myocardium. This scan was compatible with a resolving myocarditis episode, and the patient subsequently made a full recovery.

## Discussion

Cardiac disease can be associated with IBD or can be a consequence of drug side effects, with over 100 cases of pericarditis and perimyocarditis reported in the literature [7]. In fact, up to one-third of patients may develop myocarditis during the course of their disease, and this can be clinically indistinguishable from 5-ASA-induced myocarditis [8].

Myocarditis is a rare but potentially fatal adverse effect of 5-ASA therapy that usually occurs within 2–4 weeks of initiation of treatment [8]. Although the mechanism remains unclear, it is postulated that cell-mediated hypersensitivity causes myocarditis, and this is thought to be more plausible than a direct cardiotoxic effect of the drug [9]. Studies have shown that cardiac complications of 5-ASA respond well to simple discontinuation of this medication, further supporting a hypersensitivity reaction [10, 11]. However, establishing a diagnosis of myocarditis caused by 5-ASA is particularly difficult because there are no specific findings derived from laboratory or cardiac imaging that are pathognomonic of this condition [12]. The key features to recognize are the onset of chest pain, dyspnea, or fever shortly after commencing the drug, usually within 28 days. Noninvasive imaging techniques such as echocardiography and CMR have a good diagnostic yield and can offer an alternative to myocardial biopsy, which is the gold standard but is rarely performed, owing to safety reasons [13, 14]. Cessation of the drug should ensue, and other causes of myocarditis should also be excluded, including viral myocarditis, vasculitis, or an extraintestinal manifestations of IBD.

## Conclusions

The case of our patient illustrates that clinicians should be aware of this very rare but potentially life-threatening adverse effect of 5-ASA because this drug is widely prescribed for patients with IBD. It is vital to consider myocarditis in the differential diagnosis for chest pain in a patient taking 5-ASA because treatment is simply to discontinue the medication, which should improve symptoms rapidly within 24 h without leading to any permanent sequelae.

## Abbreviations

5-ASA: Mesalazine
CMR: Cardiac magnetic resonance imaging
IBD: Inflammatory bowel disease
UC: Ulcerative colitis

## References

[1] Bergman R, Parkes M. Systematic review: the use of mesalazine in inflammatory bowel disease. Aliment Pharmacol Ther. 2006;23(7):841–55. 10.1111/j.1365-2036.2006.02846.x 16573787

[2] Stelts S, Taylor MH, Nappi J, Van Bakel AB. Mesalamine-associated hypersensitivity myocarditis in ulcerative colitis. Ann Pharmacother. 2008;42:904–5. 10.1345/aph.1K288 18430794

[3] Loftus Jr EV, Kane SV, Bjorkman DJ. Systematic review: short-term adverse effects of 5-aminosalicyclic acid agents in the treatment of ulcerative colitis. Aliment Pharmacol Ther. 2004;19:179–89. 10.1111/j.0269-2813.2004.01827.x 14723609

[4] Ham M, Moss AC. Mesalamine in the treatment and maintenance of remission of ulcerative colitis. Expert Rev Clin Pharmacol. 2012;5(2):113–23. 10.1586/ecp.12.2 PMC3314328 22390554

[5] Fujiwara M, Mitsui K, Yamamoto I. Inhibition of proliferative responses and interleukin 2 productions by salazosulfapyridine and its metabolites. Jpn J Pharmacol. 1990;54:121–31. 10.1254/jjp.54.121 1981793

[6] Freeman HJ, Salh B. Recurrent myopericarditis with extensive ulcerative colitis. Can J Cardiol. 2010;26:549–50. 10.1016/s0828-282x(10)70470-0 PMC3006104 21165365

[7] Rellecke P, Strauer BE. Chronic Inflammatory bowel disease and cardiovascular complications [in German]. Med Klin (Munich). 2006;101 Suppl 1:56–60. 16802521

[8] Moss AC, Peppercorn MA. The risks and the benefits of mesalazine as a treatment for ulcerative colitis. Expert Opin Drug Saf. 2007;6:99–107. 10.1517/14740338.6.2.99 17367256

[9] Cooper Jr LT. Myocarditis. N Engl J Med. 2009;360:1526–38. 10.1056/NEJMra0800028 PMC5814110 19357408

[10] Liu Y, Ye J, Zhu J, Chen W, Sun Y. Myocarditis due to mesalamine treatment in a patient with Crohn’s disease in China. Turk J Gastroenterol. 2012;23(3):304–6. 10.4318/tjg.2012.0357 22798127

[11] Doganay L, Akinci B, Pekel N, Simsek I, Akpinar H. Mesalazine-induced myopericarditis in a patient with ulcerative colitis. Int J Colorectal Dis. 2006;21(2):199–200. 10.1007/s00384-004-0706-1 15726390

[12] Brown G. 5-Aminosalicylic acid-associated myocarditis and pericarditis: a narrative review. Can J Hosp Pharm. 2016;69(6):466–72. PMC5242279 28123193

[13] Biesbroek PS, Hirsch A, Zweerink A, *et al.* Additional diagnostic value of CMR to the European Society of Cardiology (ESC) position statement criteria in a large clinical population of patients with suspected myocarditis. Eur Heart J Cardiovasc Imaging. 2017. doi:10.1093/ehjci/jex308. [Epub ahead of print]. 29186442

[14] Dominguez F, Kuhl U, Pieske B, Garcia-Parvia P, Tschope C. Update on myocarditis and inflammatory cardiomyopathy: reemergence of endomyocardial biopsy. Rev Esp Cardiol. 2016;69(2):178–87. 10.1016/j.rec.2015.10.015 26795929

